# The Application of Deep Learning to Electroencephalograms, Magnetic Resonance Imaging, and Implants for the Detection of Epileptic Seizures: A Narrative Review

**DOI:** 10.7759/cureus.42460

**Published:** 2023-07-25

**Authors:** Arihant Singh, Vivek R Velagala, Tanishq Kumar, Rajoshee R Dutta, Tushar Sontakke

**Affiliations:** 1 Medicine, Jawaharlal Nehru Medical College, Datta Meghe Institute of Higher Education and Research, Wardha, IND

**Keywords:** magnetic resonance imaging, implants, neurosurgery, deep learning, artificial intelligence, seizures, neuroimaging, electroencephalograms, epilepsy

## Abstract

Epilepsy is a neurological disorder characterized by recurrent seizures affecting millions worldwide. Medically intractable seizures in epilepsy patients are not only detrimental to the quality of life but also pose a significant threat to their safety. Outcomes of epilepsy therapy can be improved by early detection and intervention during the interictal window period. Electroencephalography is the primary diagnostic tool for epilepsy, but accurate interpretation of seizure activity is challenging and highly time-consuming. Machine learning (ML) and deep learning (DL) algorithms enable us to analyze complex EEG data, which can not only help us diagnose but also locate epileptogenic zones and predict medical and surgical treatment outcomes. DL models such as convolutional neural networks (CNNs), inspired by visual processing, can be used to classify EEG activity. By applying preprocessing techniques, signal quality can be enhanced by denoising and artifact removal. DL can also be incorporated into the analysis of magnetic resonance imaging (MRI) data, which can help in the localization of epileptogenic zones in the brain. Proper detection of these zones can help in good neurosurgical outcomes. Recent advancements in DL have facilitated the implementation of these systems in neural implants and wearable devices, allowing for real-time seizure detection. This has the potential to transform the management of drug-refractory epilepsy. This review explores the application of ML and DL techniques to Electroencephalograms (EEGs), MRI, and wearable devices for epileptic seizure detection. This review briefly explains the fundamentals of both artificial intelligence (AI) and DL, highlighting these systems' potential advantages and undeniable limitations.

## Introduction and background

Epilepsy is any disorder where the spontaneous recurrence of unprovoked seizures is the main symptom [1]. As per an official report from the International League Against Epilepsy (ILAE), epilepsy is characterized by the occurrence of at least two unprovoked seizures happening more than 24 hours apart. It can also be defined as having one unprovoked seizure and a likelihood of experiencing additional seizures after two unprovoked seizures within the next 10 years [2]. Seizures manifest as sudden and temporary irregularities in the brain’s electrical activity, leading to disruptive symptoms. These symptoms range from momentary lapses in attention to sensory hallucinations or even full-body convulsions [3]. Epilepsy affects over 70 million people worldwide, of which around 12 million cases are in India [4]. Medically refractory epilepsy refers to the condition where seizures persist despite any medical interventions. Two distinct criteria define it. Firstly, it entails having seizure frequency occurring more frequently than every six months at the final follow-up, coupled with the failure of two or more antiepileptic drugs due to a lack of efficacy. Secondly, it encompasses cases where patients have undergone epilepsy surgery due to the failure of two or more antiepileptic drugs. Despite the implementation of multiple treatment approaches, the seizures remain uncontrolled in both scenarios [5]. Individuals who suffer from medically intractable seizures face challenges that significantly impact their quality of life, independence, and mobility [6].

Consequently, this can lead to social isolation and financial difficulties. An even more alarming aspect is that resistant seizures significantly elevate the likelihood of fractures and injury due to sudden loss of tone or convulsions, severely debilitating the person’s ability to perform specific tasks [7]. The patients may lose muscular tone abruptly, as in the case of atonic seizures, or may experience clonic or myoclonic jerky movements, as seen in generalised tonic-clonic seizures [8]. Therefore, epilepsy is not only risky when a person is driving or working with heavy machinery but might also be dangerous when a person is stationary [9,10]. Detection of epilepsy and epileptic syndromes before clinically disabling symptoms occur in patients should be of paramount importance. The interictal window period allows for early detection and intervention in patients with epilepsy. Electroencephalography (EEG) remains an important diagnostic test for epilepsy, despite the progress made in radiodiagnosis [11]. Characteristic epileptiform discharges and abnormalities during the interictal period can confirm the diagnosis and help narrow down the type of seizure [12]. 

Apart from a few exceptions, almost all individuals with epilepsy exhibit distinct changes in their EEG patterns during an epileptic seizure, also known as ictal recordings. Additionally, most epilepsy patients display specific interictal or between-seizure epileptiform discharges (IEDs) referred to as spike (<70 μsec duration), spike and wave, or sharp-wave (70-200 μsec duration) discharges [13].

## Review

Epilepsy

Epileptic seizures are episodes characterized by the hyperactivity of numerous neurons in the central nervous system. In epilepsy, neuronal excitation exceeds the inhibition of nerve impulses [14]. This imbalance can result from dysfunction in ion channels or defects in neuronal connections. Defects in connections may cause an inadequate synthesis of inhibitory neurotransmitters or the formation of abnormal excitatory associations between nerves [15]. Around 1000 genes have been associated with epilepsy [16]. The calcium channel, voltage-dependent, beta-4 subunit (CACNB4) gene is associated with juvenile myoclonic epilepsy (JME) [17]. Etiology can be difficult to identify among remote and acute causes [18]. Focal seizures originate from a local area of the cerebral cortex and exhibit clinical manifestations corresponding to that particular area [19].

In contrast, abnormal electrical activity in generalized seizures involves the entire cerebral cortex [20]. These seizures are usually accompanied by loss of consciousness, muscular rigidity, and jerking, as seen in generalized tonic-clonic seizure (GTCS), or loss of muscular tone throughout the body, as seen in atonic seizure [21]. Seizures with a focal origin, but progressing to involve the entire cortex, are secondarily generalized seizures [22].

Electroencephalograms

An electroencephalogram (EEG) scan is the primary investigation in epilepsy studies, where electrical activity is monitored using scalp electrodes. Electrodes are placed over the scalp, and the potential difference between channels is measured. Each channel denotes the activity of a specific area of the brain [23]. In generalized seizures, activity is seen in almost all scalp channels due to a generalized abnormal discharge. Activity is seen in a few channels when the seizure is focal in origin. Monitoring of epileptic seizures through EEG can be achieved manually or through automated processes. Background noise and artifacts, including eye blinks and muscle movements, may contaminate EEG signals. This interference makes visual inspection challenging in longer-duration recordings. The accuracy of diagnosis has also been inconsistent when EEG reports are read manually [24]. 

Deep learning (DL) and artificial intelligence (AI)

An automatic system that can help detect seizures in the interictal period can be beneficial for the accurate and timely diagnosis of epilepsy. Machine learning (ML) and AI concepts can help clinicians and technicians reach a diagnosis, localize the epileptic site, and effectively manage seizures. ML algorithms can examine complex EEG and imaging data to diagnose epilepsy [25]. These powerful algorithms can also help localize epileptogenic zones and predict the treatment outcomes for both medical and surgical interventions [26]. ML is a branch of AI that involves the concept of computers learning from data [27]. As a computer science and statistics discipline, ML aims to find patterns and relationships from data using efficient computer algorithms [28]. 

ML can be classified into two main types of tasks: supervised learning (SL) and unsupervised learning (UL). SL is an ML approach that begins with the objective of foreseeing a known target. Examples of SL problems include identifying graph patterns and classifying written material. SL is employed in tasks in which humans are usually proficient. It aims to approximate a human being’s performance at these tasks and stands on the pillars of classification and prediction [29]. The DL model is provided with labeled examples, from which the model learns and tries to identify patterns that differentiate the various examples. It selects the most suitable subgroup to classify the data that are provided to it. The patterns identified by the model can then be used for prediction or estimation. When used in medical diagnosis, this type of learning can not only classify the disease based on graphical/image data but also predict other factors such as the prognosis of a disease or the treatment outcome [30]. 

UL is a type of ML where the AI model learns from unlabelled data without explicit guidance or predefined outcomes. The model aims to find relationships and patterns within the data independently [31]. Instead of outputs to predict, the model tries to find naturally occurring patterns in the data in UL [32]. Even under typical scalp EEG recording setups, each electrode captures signals from the surrounding area, resulting in a noisy signal and channel crosstalk [33]. Denoising the received signals is exceptionally challenging due to the differences in volume conduction characteristics of the different structures of the head, including the skull bone, scalp, brain, and hair [34]. 

Convolutional neural networks (CNNs) and EEG

CNNs are DL models inspired by a human's nervous visual processing. These networks are competent in analyzing high-dimensional data, which may include graphs or images [35]. CNNs are multi-layered and include convolutional, fully connected, and pooling layers. All layers learn and extract hierarchical features from the input data [36]. The convolutional Layer applies filters to the input data, capturing local patterns and extracting meaningful information from the image data [37]. AI models perform artifact removal and denoising when applied to EEG data. Powerful SL models, such as support vector machines (SVM), can be used to detect anomalous data during pre-processing [38]. Data that are grossly different from usual standards are flagged at this step.

The continuous EEG signal is divided into epochs, each epoch representing specific brain activity. Epochs are labeled and then converted to spectrograms to use as input. Spectrographs are interpreted as visual representations of the frequency content of EEG signals over a period [39]. The spectrogram is seen like an image, with the frequency and time on the y and x axes, respectively, and the intensity representing the power of frequencies [40]. Relevant features and patterns are extracted from the spectrogram data, following which the spectral data are classified [41]. Much like the human brain, the data are classified according to the strength of pattern connections with pre-learned data. Epochs can be segregated as epileptic or non-epileptic, indicating the frequency and severity of these signals [42]. This is a typical use of the ML concept of SL. Once a CNN model is trained, it can be exposed to unseen EEG data [27]. 

**Figure 1:**
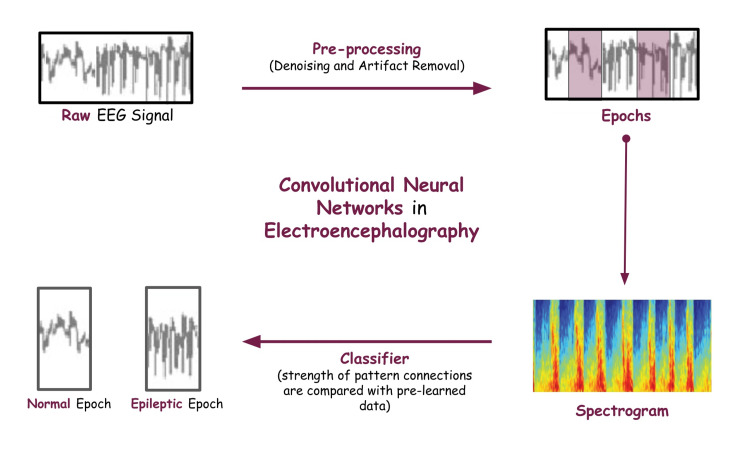
Sequence of Events of a Convolutional Neural Network (CNN) in Electroencephalography (EEG) Classification. Normal and Epileptic Epochs Have Been Classified. Image credits: Arihant Singh

CNNs have been used for various tasks in neurology, including event-related potential (ERP) analysis, epileptic seizure detection, classification of sleep stages, and brain-computer interfaces [43-45]. Recent advancements in DL techniques have shown impressive performance in various areas. Two-dimensional CNNs, such as AlexNet and very deep convolutional networks, for large-scale image recognition (VGG), and three-dimensional networks, such as 3DCNN and C3D, have shown phenomenal performance in different fields [46]. Figure 1 describes the basic sequence of events in a DL model used for EEG classification.

A recent study explored the effectiveness of CNN in differentiating temporal lobe epilepsy (TLE) from Alzheimer's disease (AD). The model achieved significant accuracy in identifying TLE cases regardless of age. Analysis revealed unique patterns associated with both diseases, mainly in temporal and extratemporal regions for TLE. The CNN model outperformed human experts detecting TLE and showed an accuracy of about 94% [47]. CNNs can therefore aid clinicians in diagnosing epilepsy and complement traditional methods. This suggests that DL and human interpretation can reduce the need for invasive procedures. Future research could extend the CNN approach to other challenges in diagnosis and optimize the model's performance. A CNN has implications for the management of epilepsy and contributes to our understanding of neuropathological changes in the condition [48].

MRI analysis and neurosurgery

Literature on ML and epilepsy has not only explored electrophysiological investigations such as EEG but also neuroimaging analysis [49]. MRI offers significant advantages in confirming the extent of the epileptic region, which is crucial for presurgical evaluation and post-surgery assessment [50]. In contrast, EEG faces challenges in accurately localizing the specific area of epilepsy-related abnormalities within the brain [51]. 

Volumetric analysis and automated segmentation of brain structures are possible using AI in MRI scans [52]. A notable project is the multi-center epilepsy lesion detection (MELD) initiative, which focuses specifically on epilepsy. This project involves collecting extensive clinical data and MRI from thousands of patients with epilepsy [53]. These datasets present a rich opportunity to apply AI techniques in epilepsy research. Integrating AI, DL, and MRI data can potentially revolutionize our perspective on epilepsy and its associated comorbidities. By optimizing the power of DL and utilizing these large datasets, it is possible for researchers to discover novel patterns, develop predictive models, and enrich our understanding of the mechanisms underlying epilepsy. The effective measurement of surgical outcomes can vastly improve surgical decision-making. 

**Figure 2:**
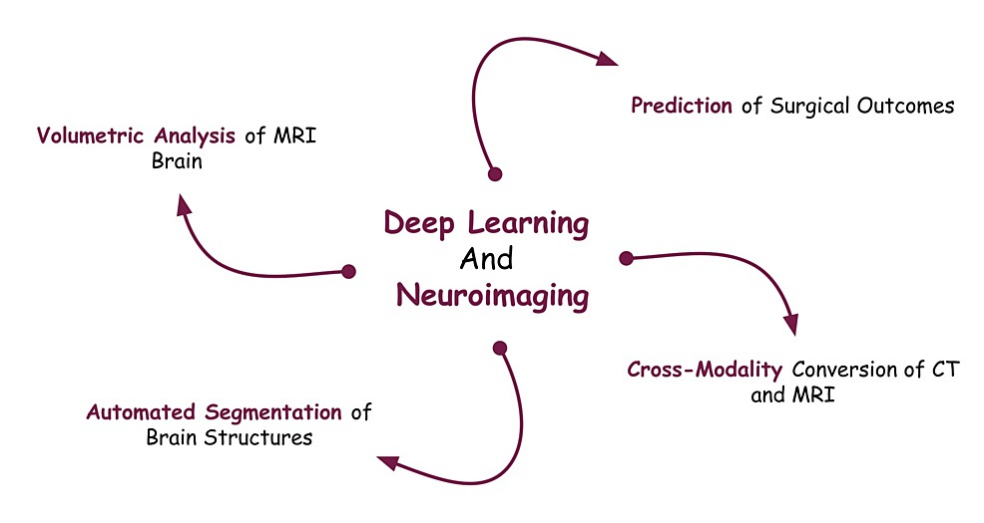
A Diagram Showing the Various Applications of Deep Learning in Neuroimaging. Image credits: Arihant Singh

ML has demonstrated remarkable performance in outcome prediction for neurosurgery. A study explored 30 evaluations of ML prediction models for patients undergoing surgery for neurological disorders, such as brain tumors, neurovascular disease, hydrocephalus, and epilepsy. Models were employed to determine recurrence, symptom improvement, and survival, where the median accuracy was found to be 94.5% with DL prediction. Compared to the traditional logistic regression model, DL models show a raw improvement in median accuracy by 15% [51]. It has also been found that cycle-consistent adversarial networks (CycleGAN) and U-Net networks can be used for the cross-modality conversion of CT and MR images [54]. Figure 2 shows the various uses of DL in neuroimaging.

In another study, a CNN was applied to assess the classification accuracy of TLE solely based on structural MRI scans. The aim was to determine whether CNN models could detect grey matter changes and aid in classifying epilepsy, focusing on TLE. The study found that CNN DL systems were comparable to human experts in identifying patients with lesional TLE. However, they outperformed human experts in identifying presumed non-lesional cases and discriminating patients from controls. The high accuracy of the CNN in classifying non-lesional TLE patients highlights the limitations of human visual classification and suggests the need for revising the concept of "lesional epilepsy." The study used 2D image classification patterns based on spatially normalized grey matter maps, but future research should explore additional features and multimodal imaging to improve classification accuracy further [55].

Wearable devices and implants 

In the realm of epilepsy detection, implanted devices that provide real-time neural activity classification can help patients by reducing the incidence of accidents during seizures [56]. The leaps made by DL algorithms in epileptic seizure defection are nothing short of extraordinary. However, the meaningful application of these techniques is possible only through capable, non-bulky hardware [57]. This hardware bottleneck makes it highly challenging to implement DL systems within low-powered wearable devices [58]. Real-time, continuous seizure prediction devices can prove to be transformative for patients of drug-refractory epilepsy [59]. The main limitations to applying DL to wearable or implantable devices include massive data computation, high power consumption, and real-time application [60]. 

In a study, a model of 45kB was evaluated across three datasets, showing a sensitivity of 99.81%. The energy consumption estimation for these small data models was less than 10mJ per interface in scalp EEG data. A power consumption of only 0.5mJ was estimated for intracranial EEG models, making implantable detection devices possible [61]. A study tackled this issue by developing and optimizing three DL models for edge deployment, focusing on seizure detection as a case study. The researchers trained a deep neural network (DNN), CNN, and long short-term memory (LSTM) network using the CHB-MIT scalp EEG database [62]. The CNN model demonstrated a sensitivity of 89.50% and a specificity of 94.86%. Another study described an algorithm using a large labeled dataset of spike data. The system's overall accuracy was 98.9% for the CNN variant and 98.6% for the fully connected neural network (FNN) variant [63]. This system had great potential for both offline and online sorting of spikes in brain-computer interface (BCI) applications. The potential future integration of DL interfaces into neural implants offers an avenue to enhance classification performance and improve therapeutic outcomes for individuals with epileptic seizures [62]. 

Limitations of ML in epilepsy detection

The application of AI and its subdisciplines in the field of seizure detection is undeniably going to shape the future of neuroscience. Effective treatment and improved medical and surgical outcomes are only possible if accurate and timely detection of seizure activity is detected. However, many limitations to the real-world applications of these systems diminish their potential. ML and DL models are known to function as “black boxes,” making it extremely challenging to interpret the reasoning behind their predictions correctly [64]. In medical applications, interpretability is crucial as accurate diagnosis and appropriate treatment depend on it. The lack of interpretability to AI models poses a significant limitation to their adoption in clinical practice. 

A recent study validated a classifier and predicted treatment response in an Australian cohort of newly diagnosed epilepsy patients. It assessed the validity of the classifier in two independent United Kingdom (UK) cohorts of newly diagnosed patients. This study investigated the predictive utility of five single-nucleotide polymorphisms (SNPs) in seizure control. It was found that the model trained on the Australian cohort could not predict the treatment response in UK cohorts [65]. Epilepsy manifests in diverse ways across individuals, making it challenging to develop models to generalize seizure activity. These models are also prone to bias, which can worsen further when trained on biased datasets [66,67]. 

**Figure 3:**
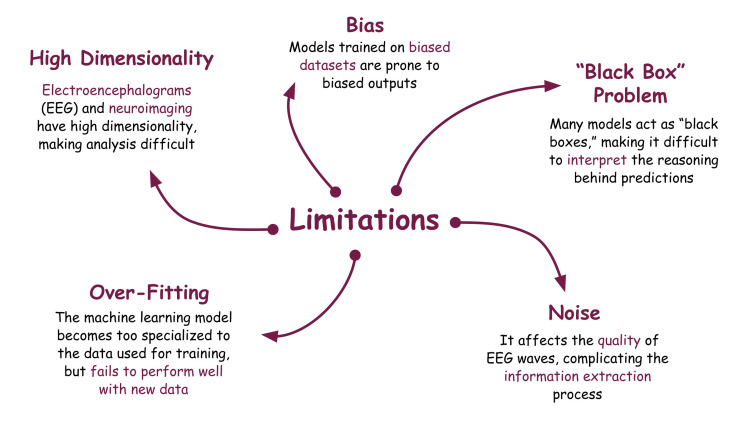
The Limitations of Deep Learning Models When Used for Epileptic Seizure Detection. Image credits: Arihant Singh

Another major limitation of DL systems is overfitting, where the ML model becomes too specialized to the data used for training but fails to perform well with new data [68]. Overfitting can result in erroneous interpretations of the results. As described above, noise can affect brain waves' quality, complicating the information extraction process. Complex preprocessing techniques must be used to enhance the signal quality and may affect the final interpretation [69]. Neurophysiological data such as EEG and imaging scans have a high dimensionality compared to the number of samples available. The reduction of dimensionality is a significant limitation and is essential to reduce the time required for training [70,71]. Figure 3 describes the current limitations of DL models in epilepsy seizure detection.

## Conclusions

In neurology, the application of DL, primarily CNNs, has significantly improved recently. CNNs can now successfully analyze EEG data by performing artifact removal, denoising, and classification of brain activity patterns. Volumetric analysis and brain structure segmentation have been made possible by applying these models to MRI data. Collaborative efforts utilizing AI techniques with large multi-center datasets, such as the MELD project, have opened new avenues for understanding epilepsy and its associated conditions. DL models have shown significant performance improvements when predicting outcomes for neurosurgery. These DL models are now surpassing the traditional logistic regression models in accuracy, which may improve decision-making related to the surgical management of epilepsy. Incorporating ML interfaces into implants and wearable devices can open new avenues for real-time seizure detection in patients with intractable epilepsy. However, for AI-detection models to be practical and effective, they should be seamlessly integrated into the clinical workflow of neurologists. Limitations such as poor interpretability hinder their adoption in accurate clinical setups. Epilepsy is a vastly complicated illness; accurate diagnosis depends on comprehending the underlying reasoning behind AI predictions. Data noise, high dimensionality, overfitting, and data bias are other limitations that are delaying the application of this versatile tool into our current diagnostic system.

In summary, CNNs models have demonstrated considerable potential in analyzing Electroencephalograms and MRI data, predicting surgical outcomes for epilepsy surgery, and in wearable device implementations. Despite the challenges posed by the limitations discussed above, ongoing advancements and research in DL will enable us to utilize these models to enhance our understanding of epileptic seizures, their detection, and their effective management.
